# Notes and News

**Published:** 1900-09-08

**Authors:** 


### Notes and News.

Dr. Alfred Greenwood, Ch.B., L.R.C.P., L.R.C.S., D.P.H., has been appointed Medical Officer of Health to the Borough of Crewe, and Medical Superintendent to the Crewe Isolation Hospital.

The Hospitals Commission in South Africa are now carrying on their inquiries at Bloemfontein. No. 8 Hospital, according to the evidence before them so far, has produced the greatest number of complaints.

Dr. James Musgrove, M.D. and F.R.C.S.Edin., has been appointed the first Professor to the new Chair of Anatomy, established and endowed by the Marquis of Bute at St. Andrews University by a gift of £20,000 for the purpose.

The Medical School of the Middlesex Hospital will re-open on October 1st, on the afternoon of which day Professor Clifford Allbutt will deliver an address to the students, and in the evening the annual dinner will be held at the Trocadero Restaurant.

There are no fresh cases of plague at Glasgow, but three persons suffering from illness are on the "doubtful" list, whilst 103 persons are under observation. All the houses infected have been cleansed and disinfected. There has been no case of death for eight days, though the 14 cases in hospital are serious. Glasgow has been declared an infected port by a large number of foreign authorities, and it is feared that its shipping trade will be affected for some time.

The Select Committee on the Exemption of Hospitals from Rating has issued its report and recommendations in a Blue Book. The recommendations are to the effect that the principle of exemption from rates should be applied to all hospitals and other institutions for the reception of sick, injured, or mentally and bodily afflicted persons, which are carried on without profit or gain, and supported for the most part from voluntary sources, and benefiting the rates of the districts in which they are situated to a larger amount than the sum at which they are rated. It is recommended that the rating authorities should have the power of exemption, and that the institutions should be able to appeal to the County Council, or some central rating authority. They also suggest a plan of distributing the loss caused by the rates which would have been paid to the districts.

The Great Western Railway Company have erected a new operating theatre at the hospital attached to their works at Swindon.

The German Malaria Commission, directed by Dr. Koch, has issued its fifth report from New Guinea. Dr. Koch holds out no hope of devising any means of exterminating the mosquito.

The foundation-stone of a new cottage hospital has been laid at Morecambe, to be called the Queen Victoria Cottage Hospital. Accommodation for eight patients in two wards will be initially provided at a cost of about £2,500.

At the recent quarterly court of the Middlesex Hospital it was announced that alterations and additions to the nurses' quarters, the wards, and the laundry are contemplated, and £1,741 was voted to be spent on cleansing and repairs.

A new cottage hospital has been opened at Wellingborough, erected through the generosity of Mr. William Woolston and Miss Mary Woolston. Miss Woolston paid for the building, and Mr. Woolston provided the site. Contributions from the neighbourhood were made for the equipment and furnishing.

A new public dispensary is to be erected at Leeds. The old buildings are too small, and they have now been encroached upon to effect the widening of North Street, where they are situated. The Corporation are paying a good price for the old premises, which they wish to acquire, so that the cost will be materially decreased. The new institution is to be on Hartley Hill, close to the former site.

The drains of the Royal Isle of Wight Infirmary have been examined, and reported as seriously defective. Such a report shows a definite danger to those residing in the institution, and the proposal to devote a sum out of the capital to remove the evil at once was only one of common sense. Yet we learn that the special meeting of governors called to take action in the matter incurred the grave responsibility of postponing action in the matter. The subscribers to the hospital should insist upon immediate steps being taken before it is too late to prevent a serious mishap.

The convalescing soldier, not unnaturally, is becoming a difficulty. The wave of enthusiastic sympathy which has turned towards Tommy Atkins in the present war has rendered the whole nation desirous that his return home should be as pleasant as possible. One consequence has been a less limited amount of furlough after illness than usual. This has told somewhat hardly upon the soldiers' friends, and it has not been generally realised that, if the convalescence of a returned hero is likely to be prolonged, he can give up his furlough and obtain the benefits of a convalescent home. If this were understood, the many disappointed people who have been preparing refuges for ailing soldiers, and finding their kindness not taken advantage of, would once more take heart. After a soldier has once visited his friends he is much less likely to object to the care and good food awaiting him elsewhere.

